# A study on the mechanical characteristics of the EBM-printed Ti-6Al-4V LCP plates *in vitro*

**DOI:** 10.1186/s13018-014-0106-3

**Published:** 2014-11-05

**Authors:** Peng-cheng Liu, Yun-ji Yang, Run Liu, He-xi Shu, Jin-peng Gong, Yong Yang, Qi Sun, Xing Wu, Ming Cai

**Affiliations:** Department of Orthopaedics, Shanghai Tenth People’s Hospital, Tongji University School of Medicine, No.301, Middle Yanchang Road, Shanghai, 200072 China; Department of Orthopaedics, Chengdu 7th People’s Hospital, No. 1 Twelve Middle Street, Wuhou District, Chengdu, 610021 Sichuan Province China; First Clinical Medical College, Nanjing Medical University, No. 140 Hanzhong Road, Nanjing, 210029 Jiangsu Province China

**Keywords:** Ti-6Al-4V, EBM, Mechanical properties, Bone plate

## Abstract

**Purpose:**

The electron beam melting (EBM) Ti-6Al-4V material technology has been developed over a short time period. It was introduced through a research to develop Ti-6Al-4V implants for patients, but EBM printed locking compression plates have not been used for clinical implants. The main purpose of this study is to find whether the EBM Ti-6Al-4V plate suit for clinical implants.

**Methods:**

First, we scanned an AO-locking compression plate (LCP) and printed LCP samples using EBM. Next, we evaluated the EBM plate surface roughness through optical microscopy as well as the LCP and EBM plates’ mechanical characteristics using the ASTM standard, which is commonly used to test the mechanical properties of bone plates subject to bending. Each sample was examined using a single-cycle four-point bending test and hardness testing to acquire data on bending stiffness, bending strength, bending structural stiffness, and hardness.

**Results:**

The results show significant differences in bending stiffness, bending strength, bending structural stiffness, and hardness between the samples using EBM and the original LCP plates. The EBM-printed samples’ surface roughness was 0.49 ± 0.02 μm. The mean hardness of the LCP sample was 266.67 HV10 ± 5.8, and the EBM-printed sample mean hardness was 341.1 HV10 ± 1.93. The EBM samples’ bending stiffness was 87.67%, which is greater than using the LCP plates’; and the bending strength was 190.7% greater, the bending structural stiffness was 73.2% greater, and the hardness was 27.9% greater.

**Conclusions:**

The results show that the EBM plates’ general mechanical strength was significantly greater than the LCP plates. An EBM plate is advantageous for clinical implants because it can be customized with great potential for improvement.

## Introduction

The locking compression plate (LCP) is part of the newest generation of AO plate fixation systems, and it yields good clinical efficacy and satisfaction according to clinical surveys [1-3]. The LCP stability depends on the angular interface between the screw and plate rather than the friction force between the plate and bone. Thus, the plate cannot contact the bone when it is implanted. On the other hand, due to the low pressure on the periosteum, the LCP has little influence on the bone blood supply. In general, LCP provides stable angulation fixation and relatively safe biological environment around the fracture area with little destruction to the periosteum or bone blood supply, which consequently, favors union with the bone [4], especially for patients with poor quality bones and delayed union or non-union [5].

Electron beam melting (EBM) is a form of 3D printing; 3D printing technology rapidly manufactures objects with various complex shapes based on a computer-aided design model or computed tomography data. Different forms of 3D printing technology and many additive processes are available. Their differences lie in the production of layered parts and materials, including selective laser melting (SLM), direct metal laser sintering (DMLS), selective laser sintering (SLS), and fused deposition modeling (FDM). Other researchers use different advanced technologies to cure the liquid material, such as stereo lithography (SLA) and laminated object manufacturing (LOM).

As a rapid manufacturing method, EBM is suitable for metal parts. The technology rapidly manufactures products with strong structures, especially implants and prostheses. Unlike traditional SLS, a high-power electron beam, rather than a laser beam, is used for EBM procedures. Because metal powder leads to light reflex during sintering, the production process is much faster. High-quality implants or prostheses are fully constructed using melted metal particles, which ensure that the products are completely void free. The full EBM process occurs in an ultra-high-vacuum environment, which prevents defects caused by oxidation. In conclusion, EBM is a reliable manufacturing technique.

Currently, the application fields for 3D printing have expanded. In orthopedic and maxillofacial surgery, implants are typically required to replace damaged or removed tissue to restore its function and appearance. However, current implants for clinical use are manufactured in a fixed pattern that does not perfectly match the patient’s defect. Especially for maxillofacial surgery, it is more difficult to generate patient-specific implants or prostheses due to the extraordinary demand in maxillofacial surgery. However, these implants can be printed in accordance with CT, MRI, or other imaging data on each patient through 3D printing technology, such as LCP, which rapidly manufactures implants that are personalized.

Previous studies have shown that materials, such as Ti-6Al-4V, can be utilized to manufacture implants or prostheses [6,7]. Cellular biomaterials fabricated using EBM have been widely used due to their good osteoinductive properties [8] and mechanical properties, as demonstrated in researches of bone tissue engineering studies. Relative tests have been conducted to measure their stiffness, and the results suggest that the mechanic behavior of the biomaterials in the cellular microstructure is similar to a cancellous bone [9]; thus, it is suitable for printing implants or prostheses using EBM for clinical applications. Harrysson et al. fabricated hip stems using Ti-6Al-4V [6]; Derand et al. also reported that a lower jawbone implant was printed through EBM [10]. Implants or prostheses for such specific human bone part typically require highly individualized customization. Because an upper extremity internal fixation system requires relatively lower mechanical strength, we printed LCP superior anterior clavicle plates using EBM. Next, we proceeded to a mechanical test to find whether the EBM Ti-6Al-4V plate suit for clinical implants. Based on the results, we may have generated a new way to manufacture plates.

## Materials and methods

### EBM-printed LCP plates

**Figure 1:**
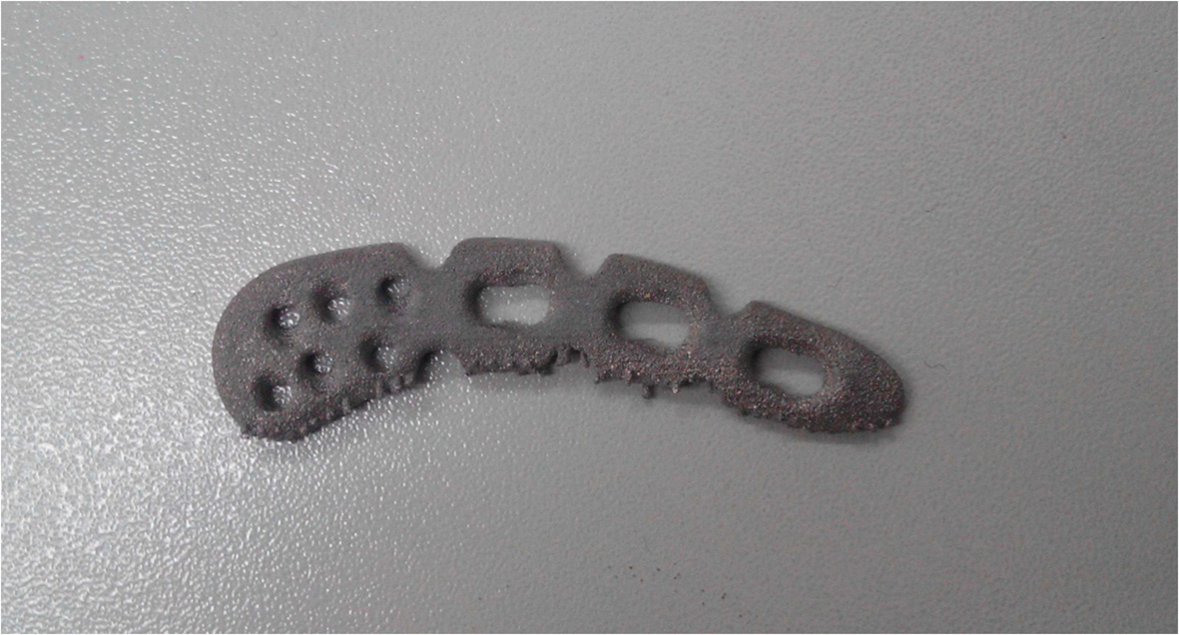
**The EBM-printed Ti-6Al-4V LCP plate.**

The LCP superior anterior clavicle plate (04.112.006; Synthes; USA) was scanned using a 3D scanner (three-dimensional sensing system; Digital Media Factory, Shanghai, China) with a 0.2-mm accuracy. Next, Geomagic Studio (Geomagic; USA) was used to import the scan as a computer-aided design (CAD) model and convert it to the *.stl file format. The stereolithography (STL) format file was inspected using Magics (Materialise; Belgium) for repair data processing. The STL file was generated without error; next, it was printed using a 3D printer (Electron Beam Melting A1; Arcam AB; Sweden). Titanium Ti6Al4V powder (Arcam AB; Sweden) was used as the printing material, and the metal powder was bombarded with a high energy electron beam layer-by-layer during the printing process; the shapes were controlled through the three-dimensional CAD using an electron beam melting system for enhanced melting and productivity. The implant can be placed in a vacuum chamber for low stress on the implant; its performance is superior to implant casting as well as forging, and the beam can be close to the implant. Printing was performed at the ambient temperature of 750°C, current I =11.5 A, and voltage V =60 KV; three samples were printed. The EBM samples are shown in Figure 1.

### Main criterion

#### Mechanical test

Three LCP plates (3 holes, 3.5 mm LCP superior anterior clavicle plates with lateral extension) and their EBM counterparts were used for the mechanical tests. For each plate, a four-point bending test was performed using the axial torsional biomechanical testing system (Shore Western 301.6, USA); the load cell was Interface 1216CEW-2 K (Interface, USA).

The plate was positioned at the bone interface, and it contacted the roller shaft surface, which positioned the plate at the bone interface and forced it to contact the roller shaft surface. Displacement-controlled testing was used at 0.1 mm/sec until plastic deformation was reached; the displacement and load data from sensors were used to establish the bending stiffness of each bone plate as defined by the ASTM standard [11] for the maximum slope in the linear elastic portion of the load versus load-point displacement curve (N/mm). The preload was 100 N. The loads were applied through the rollers with equal diameters in the range 6 mm. Each plate was positioned medially. Two screw holes were positioned between the two loading rollers. The distance between the two support rollers was 54 mm; it was 26 mm between the loading rollers. The bending moment deflection curves were generated using Microsoft Excel, and the line that represents the slope for bending stiffness was offset by a 0.052-mm displacement; this line is superimposed on the chart. The point where the bending moment-deflection curves intersects the offset bending stiffness slope is the bending strength, which is reported in N × m. The bending structural stiffness depends on bending rigidity, span center, and loading span. The computation formula used was the following: EI *e* = ((2 *h* +3*a*)*Kh* 2)/12, where ‘*K*’ is the bending stiffness, ‘*a*’ is the center span distance (26 mm), and ‘*h*’ is the loading span distance (14 mm) used to apply the controlled force and to collect the load and actuator position data.

### Secondary criteria

#### Optical microscopy and surface roughness measurement

**Figure 2:**
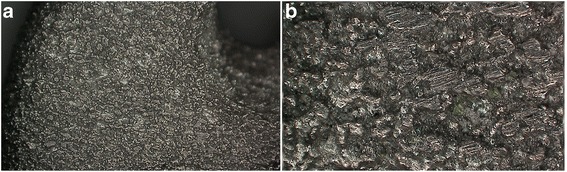
**The EBM sample surface at 35X (a) and 140X (b) magnifications.**

**Figure 3:**
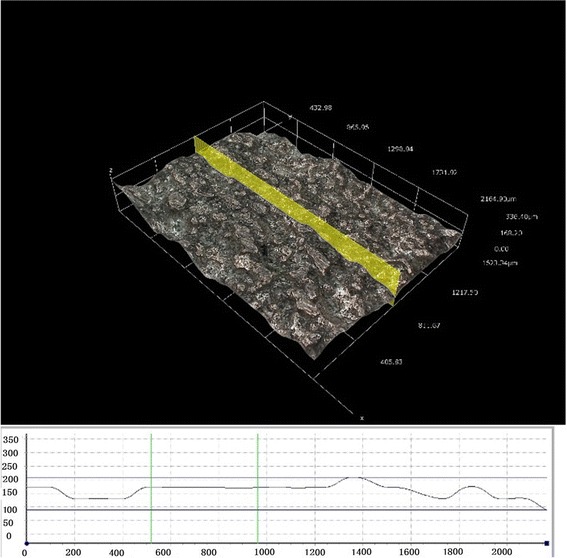
**Surface roughness measurements the length reported in μm.**

Optical microscopy was used to visualize the sample surface and record the roughness; the EBM sample surface was visualized at 35X (Figure 2a) and 140X (Figure 2b) magnifications through imaging microscopy (KH-8700 Tokyo Japan). The lens used was MXG-2500REZ. To determine the roughness and due to the sample size, the samples were indented at three different locations (Figure 3); the roughness was examined using microscopy (*n* =9).

#### Hardness test

The hardness was examined to discern the local strength of the materials at specific points, whereas the data significance for the whole material was determined using statistical means. As the most widely used method for investigating mechanical properties, examining the hardness shows each material’s differences in both chemistry and structural organization based on the manufacturing technique.

The hardness was investigated to evaluate the mechanical properties of the plates. The hardness was examined using Vicker’s test method; we examined the macro-hardness. Due to the sample size, the tests were performed at three different locations for micro-hardness testing (DHV-1000; Shanghai Material Testing Machine; Shanghai Corp; Shanghai; China). The indentations were generated with a 10-kg load and the standard indentation time was 15 s; each sample was examined (3 LCP and 3 EBM samples; *n* =18).

#### Statistical analysis

The data are expressed as the mean ± standard deviation. Two-way analyses of variance (ANOVA) were performed to detect significant differences through ROM using SPSS software (version 17.0, SPSS, IBM Corp., Armonk, NY, USA). The significance used was *P* <0.05.

## Result

Under the microscope at 35X and 140X magnifications, the observed EBM plate surface was rough with an irregular texture. The microscope was used to measure the EBM plate surface roughness (Ra =0.49 ± 0.02 μm). The EBM plate mean hardness was 341.1 HV10 ± 1.93. The mean hardness for the LCPs was 266.67 HV10 ± 5.8. The EBM plate hardness was significantly different from the LCP (*P* <0.05). The 0.2% plate offset displacement was 0.052 mm; the EBM plate yielded the variable displacement range 2027–2174 N, and the LCP yielded the plate variable displacement range 712 N–753 N. The plates did not break under the four-point bending test; the EBM printing plate and LCP plate bending areas were between the two load rollers at the roller screw. The volume, bending stiffness, bending strength, and bending structural stiffness are shown in Table (Table 1). The 3 EBM samples yielded bone plates with the strongest bending structural stiffness with the mean 1.23 N × m^2^ ±0.01; the LCP was 0.71 N × m^2^ ±0.053. The EBM-printed LCP plate bending structural stiffness significantly differed from the LCP (*P* <0.05), and it was significantly stronger than the LCP plate. The bending strengths were significantly different for the EBM samples and LCP plates (*P* <0.05); the bending stiffness values were also significantly different for the EBM samples and LCP plates (*P* <0.05).

**Table 1:** **Summary of plate hardness, bending stiffness (N/mm), bending strength (N × m), and bending structural stiffness (N × m** ^**2**^ **)**

	**Hardness**	**Bending stiffness (N × mm)**	**Bending strength (N/m)**	**Bending structural stiffness (N × m** ^**2**^ **)**
EBM sample	341.1 ± 1.93	716.32 ± 4.41	15.1 + 0.50	1.23 ± 0.01
LCP plate	266.67 ± 5.8	381.68 ± 6.52	5.194 ± 0.08	0.71 ± 0.05

## Discussion

The purpose of our study is to compare *in vitro* mechanical properties of EBM manufactured LCP samples with original LCP plates and to test their mechanical characteristics to determine whether the EBM plates meet the standard for clinical application. The samples’ *in vitro* mechanical properties were examined using the ASTM standard.

**Figure 4:**
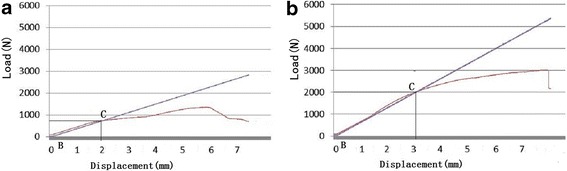
**The bone plate bending properties.** The line B-0.2% offset displacement, point C-proof load, and P (N) (the applied load at the line intersection point). The LCP maximum yield strength was 1,347 N **(a)** and the EBM plate maximum yield strength was 3,026 N **(b)**.

The results show that the EBM plate bending stiffness, strength, and structure were greater than the LCPs currently used clinically. The LCP maximum yield strength was 1,347 N (Figure 4a). Before the maximum yield strength, the LCP load versus the load-point displacement curve gently increased, while the EBM plate maximum yield strength was 3,026 N (Figure 4b). The LCP load versus load-point displacement curve is divided into three parts. In the first part, deformation and displacement are proportional to the stress. In the second part of the curve, the slope increases. The deformation and stress increase relatively slowly until the highest point. The third part, after reaching the peak, the stress falls sharply. The EBM curve exhibits a steeper slope in the first part. In the second part, the LCP curve fluctuates more dramatically compared with the EBM curve, which suggests that EBM deformation is more difficult whether it reaches the yield strength.

The same curves were reported by DeTora et al. [12], but the maximum yield strength was not reported; his LCP data differ from ours. We think that the difference may result from the different loading conditions and LCP plate types.

Eden et al. compared a seven-hole superior anterior clavicle LCP with a seven- and ten-hole reconstruction plate [13]. The clavicle LCP revealed an elastic bending stiffness with a linear slope until failure (three-point bending test). His curves were similar to ours; both the EBM (Figure 4b) and LCP (Figure 4a) plates showed a linear slope until failure in the load-to-failure test. The hardness experiment herein showed that the LCP hardness was 266.67 HV 10 ± 5.8. Peivandi et al. tested failed orthopedic implants from 23 patients; the hardness examinations showed that the implants were within the standard range 280–290 HV [14]. His result is similar to ours.

The EBM plate mean hardness was 341.1 HV10 ± 1.93, which is greater than in traditional implants.

In addition to the high strength, EBM has the advantage of custom design. LCP plates are not the only implants that EMB can produce. For example, prostheses for maxillofacial surgery and stomatology can also be manufactured using EBM technology. Recently, lower jaw or tooth prostheses were produced with various sizes. The designs are not always suitable for certain patients, but only the common type may be available, which may affect treatment [15]. Derand et al. [10] described a method for imaging via virtual design to manufacture patient-specific titanium reconstruction plates and its utility for surgically treating acquired bone defects in the mandible using additive manufacturing through EBM. For patients who require bone plates, especially elderly patients or children, EBM can provide suitable patient-specific plates.

The research of Toksvig-Larsen et al. [16] shows that the contact rate between implant and bone is only average 53%, thus easily lead to stress concentration, influence bone growth, even the bone connection looseness. So the current implants are needed for a certain degree of personalized correction, and doctors may spend a lot of time for a steel plate to meet patient skeletal shape. Personalized plate can solve this problem through the design and customization in advance. We have verified the strength of EBM printed LCP plates, it can be modified and optimized more reasonable for patients in premise to reasonable strength.

We observed the surface scratches and rough areas as well as evaluated the EBM samples’ roughness. The EBM plate surface is rough with an irregular texture, and its average roughness value was Ra =0.49 ± 0.02 μm. The implants should be polished to prevent friction between the plate and surrounding soft tissue. We also did not find the screw thread. The post process must be completed to resolve these inadequacies due to EBM inaccuracy.

EBM is also useful for porous titanium processing. To reduce its elastic modulus, designing implants with porous titanium can provide a lower bend modulus to decrease stress shielding [17,18]; implants with a porous titanium surface also facilitate bone ingrowth and are biocompatible [6,18,19]. Thus, we imagine that a porous plate surface can be designed to contact the bone, which will enhance the plate internal fixation system stability and reduce the friction between the plate and surrounding soft tissue. It may also effectively reduce the effects of stress shielding and promote bone union.

In conclusion, this study shows the mechanical superiority of EBM plates compared with the locking compression plate. The recommended testing configuration wherein the loading rollers are positioned at approximately one-third distance between the supporting rollers was not used due to the sample size. Therefore, our data and results cannot be compared with similar studies. However, we compared the EBM plates to the locking compression plate under the same conditions; the EBM plates’ general mechanical strength was significantly greater than the LCP plates, and EBM plate is advantageous because it can be customized with great potential for improvements.
